# Correlation between physical fitness, psychophysiological parameters and performance in a firearm proficiency test in military police officers

**DOI:** 10.3389/fpsyg.2025.1736902

**Published:** 2026-01-30

**Authors:** Antoniony Fantecelle Junger, Geanderson Sampaio de Oliveira, Michell Vetoraci Viana, Manuela Amaral Pinheiro, Pedro F. da C. Fortes Junior, Roberta L. Rica, Valentina Bullo, Stefano Gobbo, Marco Bergamin, Danilo Sales Bocalini

**Affiliations:** 1 Experimental Physiology and Biochemistry Laboratory, Physical Education and Sport Center, Federal University of Espírito Santo, Vitória, Brazil; 2 Department of Physical Education, Estacio de Sá University, Vitória, Brazil; 3 Department of Medicine, University of Padova, Padua, Italy

**Keywords:** firearm proficiency, military police, physical fitness, psychophysiology, training

## Abstract

**Introduction:**

Military police officers must combine physical conditioning, emotional control, and technical skills to act effectively in high-stress scenarios. However, evidence on the relationship between physical and psychophysiological indicators and performance in firearm proficiency remains scarce.

**Methods:**

This study investigated correlations between physical fitness, handgrip strength, mood state, and shooting performance among 26 military police officers of Espírito Santo, Brazil. Physical fitness was assessed by institutional protocols, handgrip strength was measured in standard and shooting positions, and firearm accuracy was evaluated under operational conditions.

**Results:**

Our data showed that overall physical fitness indicators did not correlate (*p* > 0.05) with shooting proficiency. In contrast, dominant handgrip strength (*p* < 0.05) and body mass-related parameters were positively associated with shooting accuracy and performance. Mood vigor was also linked to higher scores.

**Discussion:**

These findings suggest that targeted grip strength development and psychophysiological monitoring may represent practical strategies to optimize firearm proficiency training.

## 1 Introduction

In the state of Espírito Santo, health and public safety issues of military police officers are prominent. The occupational risks and physical and mental health problems remain high among Brazilian military police officers. A study of military police officers from different states found that police work imposes significant physical and emotional strain, associated with long hours and continuous exposure to violent situations (Arroyo et al., 2019). Similarly, more recent research indicates that occupational stress and burnout syndrome have become recurrent in this professional category, directly impacting operational performance and quality of life (Ribeiro et al., 2024). Additionally, an analysis of the conditions and equipment used in patrol activities showed that structural and operational deficiencies influence the mental health of military police officers (Barbosa Filho et al., 2025).

The response capacity of police officers in armed confrontations and high-tension situations is directly related to aspects such as muscular strength, balance, breath control, anxiety, and motor efficiency. In this context, physical and psychological preparation are fundamental to ensuring adequate performance in real conflict situations. However, there is a gap in the scientific literature regarding the correlation between physical fitness indicators and proficiency in the use of firearms, especially among military police officers.

Regarding technical-operational proficiency, the use of firearms involves a recurring action in military police activity. Its correct application, however, is not limited to technical expertise: it requires fine motor control, body stability, and focus under pressure. The Brazilian Penal Code (Brasil, 1940) provides, in Article 25, the legal foundation for self-defense as justification for the use of lethal force, which is further regulated by norms such as Interministerial Ordinance No. 4.226/2010 (Brasil, 2010) and the Basic Principles on the Use of Force and Firearms by Law Enforcement Officials (United Nations, 1990). The Military Police of Espírito Santo also establishes, in its internal normative framework, the responsible and proportional use of institutional weaponry, requiring periodic training and proficiency evaluations (PMES, 2023).

Additionally, physical fitness, within the Military Police of Espírito Santo, is considered an essential element of training and professional performance. Since the publication of Service Guideline No. 010/2014, later updated in 2023 by the Physical Fitness Test Manual, physical conditioning and periodic evaluation have become mandatory components of the daily routine for active-duty officers, aiming to promote health, reduce absenteeism, and ensure tactical readiness. In this context, research has shown that implementing structured physical training programs within police organizations contributes to improved cardiovascular capacity, muscular endurance, and operational efficiency (Massuça et al., 2022). Thus, the institutional regulations governing the Military Police of Espírito Santo also emphasize the importance of maintaining physical readiness throughout an officer’s career. Law No. 3.196/1978, in Article 9, clause IX, establishes the Physical Assessment Test (PAT) as a prerequisite for admission to the corporation, while Article 26 reinforces the duty of the military officer to maintain moral, intellectual, and physical preparedness (Espírito Santo, 1978). Such directives align with recent scientific evidence suggesting that physically active police officers present lower levels of psychological distress, reduced musculoskeletal complaints, and enhanced operational performance (Faria et al., 2024; Massuça et al., 2022). Furthermore, consistent physical training positively impacts mental health, job satisfaction, and resilience among police professionals (Rezende et al., 2022).

Complementing this theme, recent studies (Kreuzer and Natalim, 2023; Oliveira, 2024) indicate that the use of lethal force and on-duty deaths among Brazilian police officers remain high, reflecting the structural difficulties of the force and the lack of continuous and realistic training. Researchers (Simas et al., 2022; Vasconcelos Junior et al., 2025) indicate that psychophysiological variables, such as stress and handgrip strength, directly impact shooting accuracy in operational situations. These findings reinforce the need for systematic training and technical development programs geared to the operational realities of Brazilian police forces (Smith and Boolani, 2024; Borba, 2022).

Therefore, maintaining an institutional culture that prioritizes continuous physical training and health monitoring is fundamental to sustaining both the efficiency and well-being of military police officers. These findings reinforce the role of physical fitness not only as a tactical requirement but also as a determinant of occupational health and professional longevity in police service.

According to recent research, many shots fired in armed confrontations miss the target or fail to neutralize the threat effectively, raising questions about the relationship between police officers’ physical conditioning and their technical skill in firearm handling (Vasconcelos Junior et al., 2025; Simas et al., 2022). Studies demonstrate that physiological and psychological factors — such as handgrip strength, stress level, and fatigue — directly affect aim stability and shooting accuracy, reinforcing the importance of regular physical training and stress-management strategies (Simas et al., 2022; Vasconcelos Junior et al., 2025). The physical and psychological demands placed on military police officers are aggravated by the urban violence scenario and the need for rapid, high-stakes decision-making. Recent evidence also confirms that exposure to stress and long-term operational strain can impair motor performance and increase occupational risk (Pelegrini et al., 2018).

Given this context, the present research aims to analyze the correlation between physical and psychophysiological parameters and the performance in firearm proficiency among military police officers of Espírito Santo. It seeks to understand whether certain components of physical fitness and psychophysiological state directly influence shooting performance, with the intention of supporting the development of more effective training programs aimed at the training and technical-operational improvement of these professionals. Given the inconsistencies and the need for studies dedicated to investigating shooting accuracy parameters, as well as understanding how certain parameters correlate with proficiency in police shooting, the objective of this study was to correlate physical fitness, psychophysiological parameters, and the performance of firearm proficiency in military police officers of Espírito Santo.

## 2 Materials and methods

### 2.1 General characteristics and sample selection

After approval from the Research Ethics Committee in Humans of the Federal University of Espírito Santo (No. 6.275.609/2023, CAAE: 70736323.2.0000.5542), and authorization from the Directorate of Education of the Military Police of Espírito Santo (DMPES), military police officers of both sexes from the Officer Training Course of the Military Police of Espírito Santo (MPES) were invited to voluntarily participate in the study. The participants, after consenting, signed a Free and Informed Consent Form, in accordance with the norms established in Resolution No. 466/2012 of the National Health Council.

The invitation to participate in the study was made through direct contact with the military personnel and by verbal and digital dissemination strategies. As a non-inclusion criterion, military police officers on leave from work for any reason during the study period, as well as the non-signing of the free and informed consent form, were used. Military personnel who did not respond to or participate in all collections and analyses of the study were excluded. After applying the inclusion, non-inclusion, and exclusion criteria, 26 military police officers were evaluated with a mean age of 31.04 ± 2.66 years, being 5 (19%) women and 21 (81%) men with an average professional experience of 7.71 ± 3.75 years of work from the Officer Training Course of MPES.

Thus, the present research represents an important initial contribution to the relationship between physical fitness indicators and proficiency in the use of firearms among MPES, providing consistent preliminary data that can guide training strategies, occupational health, and functional performance of these professionals, in addition to guiding future work with larger samples and complementary approaches.

### 2.2 Data collection design

The Officer Training Course of MPES is the initial and essential stage for entering the career of a combatant officer, aimed at preparing leaders and managers in public security. Held at the Military Police Academy of Espírito Santo (MPAES), on a full-time basis and lasting 3 years, the Officer Training Course is a bachelor’s degree course in Police Sciences and Public Security that combines technical, legal, operational, and managerial training. The course workload extends over three academic years and is delivered at MPAES, with curricular components covering the core operational, legal, management, and ethics domains (PMES, 2024; PMES, 2024). During the course, military police officers face an intense routine of studies, physical and practical training, supervised internships, and participation in institutional activities, focusing on the development of competencies such as leadership, decision-making under pressure, discipline, ethics and involves an average of 3 years of preparation (PMES, 2024; PMES, 2022).

The data collection stage took place on the premises of the MPAES, during working days and according to the availability of the participants at last month of course. Initially, the volunteers underwent anthropometric measurements and handgrip strength tests, wearing the specific uniform for military physical training. Subsequently, they answered self-report instruments that addressed aspects related to perceived stress levels and habitual physical activity practice. On the following day, the activities were conducted at the MPAES’s own shooting range. Wearing the operational uniform and using regulatory personal protective equipment—such as a ballistic vest, safety glasses, and hearing protection—the participants were again instructed on the technical procedures for shooting. In the afternoon, they were subjected to the shooting accuracy assessment, as part of the “Police Shooting III” discipline, a curricular component of the Officer Training Course (PMES, 2024; PMES, 2022).

## 3 Parameters assessed

### 3.1 Anthropometric measurements and body composition

For the anthropometric assessment, body weight (Marte Scientific digital scale, LS200P; accuracy 0.1 kg) and height (Cardiomed stadiometer, model WCS; accuracy 0.1 cm) were measured. The body mass index (BMI) was calculated as body mass divided by height squared (kg/m^2^). The nutritional status was classified as follows: Normal (> 18.5, < 25.0), Overweight (≥ 25.0, < 30.0), and Obesity (≥ 30.0, < 40.0), according to the updated recommendations of the World Health Organization (WHO, 2020).

Body composition was estimated by a doubly indirect method using skinfold measurements at the bicipital, tricipital, subscapular, and suprailiac sites (Mitutoyo plicometer, Cescorf, Porto Alegre, Brazil; accuracy 0.1 mm). The percentage of body fat (%BF) was estimated according to Durnin and Womersley (1974), and classified as: normal (< 18%), overweight (18–24.9%), and obese (> 25%). Waist circumference (WC) was measured with an inelastic tape at the midpoint between the lower rib border and the iliac crest. For cardiovascular risk classification, increased risk was defined as ≥ 94 cm for men and ≥ 80 cm for women, and substantially increased risk as ≥ 102 cm for men and ≥ 88 cm for women (WHO, 2020). The abdomen circumference was measured at the umbilical level with the relaxed abdomen. For the hip circumference, greater apparent circumference of the gluteus was used with the tape in the transverse plane. The measurements were made in triplicate and the median values were adopted.

The waist-to-height ratio (WHtR) was calculated by dividing WC by height (cm), using 0.50 as the cutoff for increased cardiometabolic risk (Corrêa et al., 2019). Integrating these anthropometric indicators with physical performance assessments provides a comprehensive profile of health, fitness, and operational readiness in police officers.

### 3.2 Physical activity time

Total weekly physical activity (PA) time was utilized to classify the PA level according to the International Physical Activity Questionnaire (IPAQ-short version), as applied in other studies (Oliveira et al., 2024; Vasconcelos Junior et al., 2025). The questionnaire explored the frequency and duration of physical activities, including walking and moderate and vigorous exercise. Military personnel were considered active or meeting physical activity (PA) recommendations if they achieved 150 min or more PA per week; those who did not meet this threshold were classified as inactive.

### 3.3 Physical fitness assessment

The physical fitness assessment of the military police participating in this study was carried out on February 7 and 9, 2023, in accordance with the official protocols established be MPES for the application of the Physical Fitness Test (PFT). The procedure followed the standards described in the Physical Fitness Test Application Manual (PMES, 2023), approved by Ordinance No. 1070-R, dated April 27, 2023, and published in the MPES General Bulletin No. 018 on the same date.

The PFT is composed of five tests designed to evaluate the essential physical abilities for operational readiness: 2400-meter run, agility test (shuttle run), pull-ups, push-ups, and rower sit-ups. Assessments were conducted in adequately prepared military facilities to ensure participant safety and data reliability, following the technical guidelines of the corporation (PMES, 2023).

The 2,400-meter run was performed on an athletics track, allowing alternating running and walking according to individual conditioning and used the time to complete the test. The agility test followed the standardized shuttle-run protocol over 9.14 meters, where the shortest time of two trials (with 2-min intervals) was recorded and the time to complete the test. In the pull-ups test, participants began from a full-hang position (pronated grip) and performed repetitions until the chin cleared the bar. In the pull-ups test, participants began from a full-hang position (pronated grip), pulling up until the chin cleared the bar, counting the maximum number of repetitions in 1 min. The rower sit-ups involved simultaneous trunk and leg flexion–extension, counting the maximum number of repetitions in 1 min. All tests were administered by trained evaluators under institutional supervision, ensuring compliance with MPES standards and reproducibility of results.

These procedures align with national and international recommendations for police physical readiness, emphasizing the role of regular evaluation in operational performance and occupational health (Rezende et al., 2022; Carassai, 2023).

### 3.4 Handgrip strength

The assessment of handgrip strength, measured in kilograms (kg), was carried out using a Jamar manual dynamometer (Jamar® Smart Hand Dynamometer, Plus model, USA) in two positions: standard position (elbow at 90°) and in a shooting position freely adopted by the volunteer, as studied previously by Vasconcelos Junior et al. (2025). Briefly, for the standard position, the volunteers were instructed to remain seated in a chair (without arms) and with an erect spine, maintaining a knee flexion angle of 90°, the shoulder positioned in adduction and neutral rotation, the elbow flexed at 90°, with the forearm in mid-pronation and neutral wrist, being able to move it up to 30° of extension. The arm was kept suspended in the air with the dominant hand positioned on the dynamometer, being supported by the evaluator, as established by Fess and Moran (1981). The same procedure was performed for the non-dominant hand. For the evaluation of the shooting position, the military personnel were instructed to assume their usual posture used during shooting, using the dominant hand, as utilized by Vasconcelos Junior et al. (2025).

### 3.5 Strength of the lumbar extensor and shoulder girdle musculature

The lumbar extensor musculature was evaluated using a hydraulic lumbar dynamometer (Crown brand hydraulic lumbar dynamometer, USA), with a capacity of 200 kgf and divisions of 1,000 kgf according to Cavazzotto et al. (2012). To perform the test, the volunteer stood on the platform with knees and elbows fully extended and the trunk semi-flexed until the hands could hold the apparatus bar. After correct positioning, the subject was instructed to perform a maximal voluntary isometric contraction in order to extend the trunk, causing the force to be exerted by the lumbar region.

The shoulder girdle musculature was evaluated using a scapular dynamometer (Crown brand hydraulic lumbar dynamometer, USA), with a capacity of 50 kgf and divisions of 500gf in accordance with Turner et al. (2009). The evaluation with this dynamometer occurred with the volunteer in an orthostatic position, with feet apart, trunk erect, head directed forward, shoulders abducted at 90°, holding the scapular dynamometer with both hands at the same time and using all fingers, including the thumb through movement scapular adduction or shoulder horizontal abduction.

For lumbar extensor and shoulder girdle musculature, all protocols were standardized consisting of two maximum attempts, with a 1 min rest interval between attempts. The tests were performed in the order described, and once performed, the next test was immediately started, as different muscle groups were required.

### 3.6 Mood state

Mood was assessed using the Brunel Mood Scale (BRUMS), derived from the Profile of Mood States (POMS), in concordance with previous studie (Beedie et al., 2000; Lane and Terry, 2000). Mood is recognized as an important marker of transient emotional state, directly affecting concentration, fine motor control, risk perception, and reaction time—skills essential for the precise and safe execution of firearm use. Variations in subscales such as fatigue, tension, confusion, or anger can compromise self-control and motor stability, consequently impairing shooting accuracy.

Studies (Beedie et al., 2000; Lane and Terry, 2000) indicate that the so-called “iceberg profile”—characterized by high vigor and low levels of tension, depression, anger, fatigue, and confusion—is associated with positive emotional states and superior performance in activities that demand precision and emotional control under stress (Beedie et al., 2000; Lane and Terry, 2000). In this sense, monitoring the mood state of police officers in training and operational contexts provides valuable insights into fluctuations in technical performance, contributing to the development of integrated strategies for physical, psychological, and emotional readiness in military organizations.

The BRUMS questionnaire evaluates six affective dimensions: tension, depression, anger, vigor, fatigue, and confusion. The negative mood factors (tension, depression, anger, fatigue, confusion) are contrasted with the positive factor (vigor). The total mood disturbance (TMD) was calculated as the sum of negative factors minus the vigor score (TMD = [T + D + A + F + C] – V), with an additional 100 points added to avoid negative totals. Participants were instructed to respond according to how they were feeling “right now, at this very moment.”

### 3.7 Shooting procedure

The Defensive Shooting for the Preservation of Life (DSPL), known as the Giraldi Method, was developed by Colonel Nilson Giraldi from São Paulo Military Police with the aim of training military police officers to use their firearms in a technical, tactical, and psychological manner, respecting the principles of legality and human rights. The method emphasizes the preservation of life as a fundamental principle, ensuring that lethal force is used only as a last resort, in accordance with current legislation and international human rights treaties. DSPL is based on the creation of positive conditioned reflexes, acquired through realistic training that simulates operational situations, while seeking to eliminate negative reflexes. In this way, the police officer is prepared to respond in a controlled and efficient manner in high-stress scenarios, reducing the probability of unnecessary shots and ensuring the safety of all involved – including the officer themselves, innocent citizens, and even individuals in a criminal situation, in cases where neutralization by shooting is not necessary.

The DSPL proficiency assessment takes place at the end of the shooting fundamentals training, where the military personnel are subjected to a practical evaluation, in which they perform 10 (ten) firearm shots at a distance of 5 (five) meters from the target, in different shooting positions, according to the standard dynamics of the method as described in Vasconselos Junior et al., (2025). The target used has dimensions of 80×54 cm, with the central (gray) zone being 50×32 cm. Only hits in the gray zone are considered valid, while shots in the white zone (adjacent area) are not counted as hits. The sequence of shots follows the protocol in which the participant peforms two shots in the standing position, two in the kneeling position, two in the crouching position, two in the prone position, and finally, two in the standing position (Giraldi, 1998). The military personnel were instructed to fire ten shots towards the target located five meters away, without time control, in a standardized lighting environment, wearing standard operational uniform, according to the recommendations of Thomas et al. (2018) and Ito et al. (1999), to minimize external influences on shooting performance.

The accuracy assessment was based on the score obtained according to the impact position of the shots on the target: 3 points for shots that hit the center of mass; 2 points for shots in the upper region, but outside the center; 1 point for shots that hit the extremities. The task execution time and the final score were recorded, with the performance being calculated by the ratio between the total score and the task execution time. This criterion allows for the evaluation of not only the officer’s accuracy but also their ability to maintain shooting efficiency under pressure and in different postures.

### 3.8 Statistical analysis

Data are presented as absolute (n) and relative (%) frequencies for qualitative variables, and as mean ± standard deviation (SD), coefficient of variation (CV), and 95% confidence interval (95% CI) for quantitative variables. After verifying data normality using the D’Agostino–Pearson omnibus test (D'Agostino and Pearson, 1973). Pearson’s correlation coefficient (r) was used to determine the strength and direction of relationships between anthropometric variables, work time, and sleep duration. The strength of correlation was classified as weak (r = 0.10–0.30), moderate (r = 0.40–0.60), and strong (r = 0.70–1.00) (Dancey and Reidy, 2017). The effect size was calculated using Hedges’ g, interpreted as small (0.2–0.5), moderate (0.5–0.8), or large (> 0.8) (Lakens, 2013). All analyses were performed using GraphPad Prism software (version 6.00 for Windows; GraphPad Software, La Jolla, CA, USA). Statistical significance was set at *p* < 0.05.

## 4 Results

The anthropometric measurements and body composition parameters of military police officers are described in Table 1. In general, 19 (67%) of the military personnel were classified as overweight, 6 (23%) as normal weight, and 1 (4%) as obese by BMI. Twenty three (88%) of the military personnel were classified with a normal body fat percentage, and 3 (12%) were classified as overweight. Similar data were found for cardiovascular risk assessed by waist circumference and waist-to-hip ratio, with 25 (96%) of the military personnel showing no risk and 1 (4%) moderate risk. Considering the data on the waist-to-height ratio, 22 (85%) of the military personnel had a low risk and 4 (15%) a high risk.

**Table 1:** Anthropometric measurements and body composition parameters of military police officers.

Parameters	Mean ± SD	CV (%)	95% CI
Body mass (kg)	80.85 ± 10.50	13.09	76.10–84.41
BMI (kg/m^2^)	25.81 ± 2.44	9.47	24.84–26.77
Fat body (%)	12.84 ± 3.35	26.09	11.52–14.17
Fat mass (kg)	10.36 ± 3.28	31.72	9.05–11.66
Fat free mass (kg)	69.89 ± 9.05	12.95	66.31–73.47
Muscle mass (kg)	38.86 ± 5.07	13.06	36.71–41.00
Waist circumference (cm)	82.33 ± 6.23	7.56	79.70–84.96
Waist-to-height ratio	0.82 ± 0.05	7.20	0.79–0.84

Values are expressed as mean ± standard deviation (Mean ± SD), coefficient of variation (CV), and 95% confidence interval (95% CI).

As shown in Table 2, the physical activity time, physical fitness, and mood state parameters of the military police officers indicated that they were physically active, with high physical fitness and suggestive of an adequate mood state.

**Table 2:** Physical activity times, physical fitness and mood state parameter of military police officers.

Parameters	Mean ± SD	CV (%)	95% IC
Physical activity time			
Light physical activity (min)	158.50 ± 148.30	93.61	98.55–218.40
Moderate physical activity (min)	166.20 ± 145.50	87.55	107.40–224.90
Vigorous physical activity (min)	158.20 ± 161.40	102.0	93.02–223.40
Total physical activity (min)	482.80 ± 355.60	73.64	339.20–626.5
*Physical fitness*			
Push up (reps)	48.12 ± 4.06	8.44	46.47–49.76
Pull up (reps)	14.19 ± 3.46	24.41	12.79–15.59
Rower sit-ups (reps)	55.65 ± 4.16	7.48	53.97–57.34
Agility (sec)	9.20 ± 0.36	3.97	9.06–9.35
2,400 meter run (sec)	561.00 ± 46.89	8.35	542.00–579.90
*Mood state*			
Tension	3.50 ± 2.56	73.29	2.46–4.53
Depression	1.96 ± 2.39	121.90	0.99–2.92
Angry	3.34 ± 4.34	129.80	1.59–5.10
Vigor	5.42 ± 2.81	51.94	4.28–6.56
Fatigue	6.80 ± 4.34	63.83	5.05–8.56
Mental confusion	1.38 ± 2.29	166.10	0.45–2.31
Mood disturbance	111.60 ± 13.23	11.86	106.20–116.90

Values are expressed as mean ± standard deviation (Mean ± SD), coefficient of variation (CV), and 95% confidence interval (95% CI).

**Figure 1:**
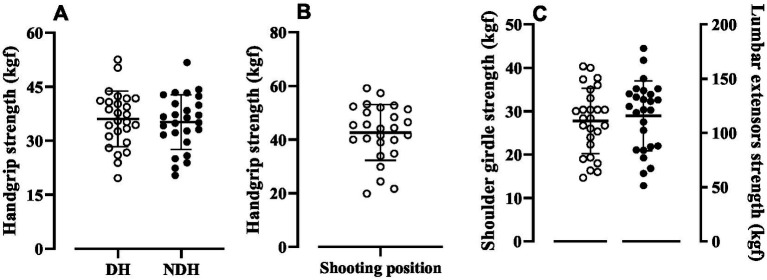
Handgrip strength of dominant hand (DH) and non-dominant hand (NDH) **(A)**, handgrip strength in shooting position **(B)** and shoulder girdle and lumbar extensor strength **(C)**. Values are expressed as mean ± standard deviation.

No difference was found in handgrip strength between the dominant and non-dominant limbs (Dominant: 36.07 ± 7.71 [CV: 21.39%] kgf, Non-dominant: 35.17 ± 7.57 [CV: 21.53%] kgf; MD: -0.903; 95%CI: −2.769 – 0.9614; ES: 0.1731; *p* = 0.3278) as showed at Figure 1A. The handgrip strength with the dominant limb in the shooting position (Figure 1B) was 42.20 ± 9.96 (CV: 23.61%) kgf. The isometric forces of the shoulder girdle and lumbar extensor muscles (Figure 1C) were 27.76 ± 7.52 (CV: 27.11%) kgf and 115.75 ± 32.19 (CV: 27.81%) kgf, respectively.

**Figure 2:**
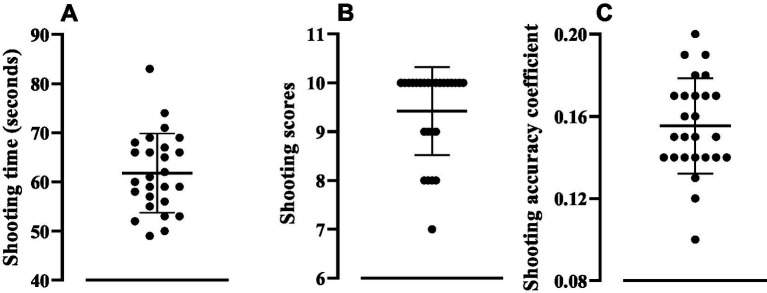
Shooting time **(A)** shooting score **(B)** and shooting accuracy coefficient **(C)**. Values are expressed as mean ± standard deviation.

In the Figure 2 is showed the shooting parameters. The shooting time (Panel A) was 61.81 ± 8.03 (CV: 13.00%) seconds, the average score (Panel B) was 9.42 ± 0.90 (CV: 9.57%) points, with a shooting performance (Panel C) of 0.15 ± 0.02 (CV: 14.93) points/min; after normalization, the values were 7.76 ± 1.16 (CV: 14.93).

As showed in Table 3 correlations between time, score, and shooting performance with anthropometric parameters, physical activity time, handgrip strength, mood state, and physical fitness of the military personnel were found.

**Table 3:** Correlation between time, score, and performance of shots with anthropometric parameters, physical activity time, handgrip forces, mood state, and physical fitness of the military police officer.

Shooting									
Parameters	Time			Score			Shooting coefficient		
	r	IC 95%	*p*	r	IC 95%	*p*	R	IC 95%	*p*
Body mass (kg)	−0.133	−0.509–0.284	= 0.532	0.410	0.008–0.698	= 0.046	0.412	0.010–0.699	= 0.045
BMI (kg/m^2^)	−0.283	−0.616–0.135	= 0.180	0.319	−0.096–0.640	= 0.128	0.454	0.062–0.724	= 0.025
Fat body (%)	−0.087	−0.474–0.327	= 0.684	0.122	−0.295–0.501	= 0.568	0.279	−0.139–0.613	= 0.185
Fat mass (kg)	−0.163	−0.532–0.256	= 0.444	0.279	−0.139–0.613	= 0.186	0.435	0.039–0.713	= 0.033
Fat free mass (kg)	−0.095	−0.480–0.320	= 0.656	0.376	−0.031–0.676	= 0.069	0.320	−0.095- 0.640	= 0.127
Muscle mass (kg)	−0.065	−0.456–0.347	= 0.760	0.309	−0.107–0.633	= 0.140	0.231	−0.189–0.580	= 0.275
Waist circumference (cm)	−0.173	−0.539–0.246	= 0.416	0.296	−0.121–0.624	= 0.160	0.342	−0.070–0.655	= 0.101
Waist-to-height ratio	−0.250	−0.593–0.170	= 0.238	0.145	−0.273–0.518	= 0.496	0.278	−0.141–0.612	= 0.188
Waist-to-hip ratio	−0.191	−0.552–0.229	= 0.370	0.013	−0.392–0.414	= 0.951	0.128	−0.290–0.505	= 0.551
Light physical activity (min)	0.237	−0.165–0.572	= 0.243	−0.026	−0.409–0.364	= 0.897	−0.217	−0.558–0.185	= 0.284
Moderate physical activity (min)	0.227	−0.175–0.564	= 0.264	0.134	−0.266–0.496	= 0.511	−0.120	−0.484–0.280	= 0.559
Vigorous physical activity (min)	0.011	−0.377–0.397	= 0.955	0.149	−0.252 – 0.507	= 0.465	0.039	−0.353 – 0.420	= 0.848
Total physical activity (min)	0.197	−0.205–0.543	= 0.334	0.112	−0.287–0.478	= 0.585	−0.122	−0.486–0.278	= 0.552
Handgrip strength of dominant	0.050	−0.344–0.429	= 0.808	0.504	0.127–0.754	= 0.011	0.186	−0.216–0.535	= 0.362
Handgrip strength in shooting position	0.108	−0.291–0.475	= 0.598	0.431	−0.033–0.710	= 0.003	0.211	−0.192–0.553	= 0.300
Shoulder girdle strength	0.163	−0.239–0.518	= 0.425	0.262	−0.139–0.589	= 0.196	−0.022	−0.406–0.368	= 0.914
Lumbar extensor strength	0.087	−0.310–0.459	= 0.671	0.213	−0.189–0.555	= 0.294	0.019	−0.370–0.403	= 0.925
Tension	−0.042	−0.422–0.350	= 0.836	0.087	−0.310 – 0.459	= 0.669	0.143	−0.257 – 0.503	= 0.483
Depression	0.118	−0.281–0.483	= 0.563	0.125	−0.275–0.489	= 0.540	−0.008	−0.394–0.379	= 0.965
Angry	0.137	−0.264–0.498	= 0.503	0.159	−0.243 – 0.514	= 0.437	−0.036	−0.418 – 0.355	= 0.859
Vigor	0.046	−0.347–0.426	= 0.821	0.438	0.061–0.706	= 0.025	0.332	−0.063–0.637	= 0.097
Fatigue	0.095	−0.302–0.465	= 0.641	0.232	−0.169–0.568	= 0.252	0.158	−0.244–0.514	= 0.440
Mental confusion	−0.130	−0.493–0.270	= 0.524	0.116	−0.283–0.482	= 0.571	0.261	−0.140–0.589	= 0.197
Mood disturbance	0.057	−0.337–0.435	= 0.781	0.095	−0.303–0.465	= 0.643	0.040	−0.352–0.421	= 0.842
Push up	−0.034	−0.416–0.357	= 0.865	0.382	−0.005–0.670	= 0.053	0.265	−0.135–0.592	= 0.189
Pull up	−0.057	−0.435–0.337	= 0.780	0.227	−0.175–0.565	= 0.263	0.169	−0.233–0.522	= 0.408
Rower sit-ups	0.252	−0.149–0.582	= 0.213	0.305	−0.092–0.619	= 0.128	−0.037	−0.418–0.355	= 0.857
Agility	0.045	−0.347–0.425	= 0.824	−0.072	−0.447 – 0.323	= 0.724	−0.079	−0.453 – 0.317	= 0.699
2,400 meter run	−0.141	−0.501–0.260	= 0.490	−0.211	−0.553 – 0.191	= 0.299	0.029	−0.362 – 0.411	= 0.887

## 5 Discussion

Within the spectrum of professional occupations, the military police career stands out for its high degree of physical, emotional, and technical demand, as well as the adverse conditions to which its members are frequently exposed. The literature highlights that the nature of this profession involves constant risks (Pelegrini et al., 2018), frequent confrontations with violent situations, decision-making under pressure, and vulnerability to psychological distress, which makes it one of the occupations most susceptible to occupational stress (Benevides-Pereira, 2010). The use of lethal force and on-duty deaths among Brazilian police officers remain high, reflecting the structural difficulties of the force and the lack of continuous and realistic training (Kreuzer and Natalim, 2023; Oliveira, 2024). Research (Simas et al., 2022; Vasconcelos Junior et al., 2025) also indicates that psychophysiological variables, such as stress and handgrip strength, directly impact shooting accuracy in operational situations. These findings reinforce the need for systematic training and technical development programs geared to the operational realities of Brazilian police forces (Smith and Boolani, 2024; Borba, 2022).

Empirical evidence reinforces the importance of structured and continuous physical training for police officers. Studies have demonstrated that regular participation in physical conditioning programs results in significant improvements in cardiorespiratory fitness, muscular endurance, and operational performance (Rezende et al., 2022; Carassai, 2023). Specifically, Rezende et al. (2022) found that Brazilian military police officers engaged in ongoing training exhibited superior physical performance indicators compared to those in administrative roles. Similarly, Carassai (2023) showed that adherence to institutional fitness standards correlates with enhanced performance in official proficiency assessments, underscoring the value of consistent physical conditioning for occupational readiness.

The sample in this study, composed of military police from the Officer Training Course of the MPES, showed a predominance of male participants—a finding consistent with other studies on Brazilian military police officers (Rezende et al., 2022). This prevalence may be related to historical and sociocultural factors, as female participation in military forces remains proportionally lower nationwide. However, the current Officer Training Course of MPES admission notice does not establish any gender distinction in available positions, indicating that such predominance stems from external factors rather than administrative restrictions (PMES, 2024).

The evaluated police officers were in the final stage of their training — a period characterized by high theoretical, physical, and psychological demands. This stage involves intense operational training, academic assessments, and field activities that increase occupational stress and emotional overload (Jacinto, 2022). Previous research has shown that elevated stress levels in military and law-enforcement contexts can impair decision-making, attention, and motor performance, particularly under pressure or threat (Baldwin, 2022; Taverniers et al., 2011).

In this study, participants presented relatively high fatigue scores and a mean total mood disturbance - values comparable to those reported in athletes exposed to demanding physical and psychological conditions (Parsons-Smith et al., 2022). This pattern resembles the so-called “iceberg profile,” characterized by high vigor and low scores on negative mood dimensions (Beedie et al., 2000; Lane and Terry, 2000). Such a profile has been associated with better performance under pressure, suggesting that despite high demands, the police officers of present study demonstrated adequate emotional regulation and coping capacity — likely enhanced by regular physical training and the disciplined routines of military education.

From an anthropometric standpoint, the police officers had a mean body mass index, placing them predominantly in the overweight category. However, body-composition analysis revealed an average body-fat percentage which is considered excellent for active populations, especially within the military context. These findings highlight the limitation of BMI as an isolated indicator among individuals with high muscle mass (Itacarambi et al., 2019; Minayo et al., 2011). Similar results have been reported in studies linking structured physical-training programs to improvements in body composition and fitness levels in military police (Rezende et al., 2022; Santos et al., 2021).

In summary, the results suggest that police officers of MPES present a balanced psychophysiological profile, with adequate physical and emotional preparedness for operational demands. However, fluctuations in mood and fatigue indicators underscore the importance of continuous institutional monitoring of both mental and physical health throughout the training process — promoting sustainable performance and long-term well-being.

The prevalence of 88% of participants with a body fat percentage within the normal range, combined with the low cardiovascular risk indices observed in the indicators of waist circumference, waist-to-hip ratio, and waist-to-height ratio, reinforces the need to adopt a multidimensional approach for the assessment of nutritional status and physical fitness in military police officers. Our results suggest that, although the BMI indicates overweight in 67% of the sample, the indicators of body composition and metabolic health - such as body fat percentage, waist circumference, and lean mass - point to a picture compatible with good physical fitness, which requires caution when using BMI alone for the diagnosis of overweight or obesity in this group. It is believed that the low body fat percentage in our sample can be explained by the balanced diet provided in the institutional cafeteria, as well as the high volume of physical activity required throughout the training course, which typically involves an average of three years of preparation.

Thus, it is important to highlight that we found a high degree of involvement with weekly physical activity. Similar to others studies from our group with military police officers from the specialized policing of Espírito Santo (Sampaio et al., 2024) and in officer training (Vasconcelos Junior et al., 2025), we demonstrated an high involvement with physical activity practice per week. It is worth mentioning that these values are considered substantially higher than the minimum recommendations established by the WHO (2020), which suggest at least 150 to 300 min of moderate physical activity or 75 to 150 min of intense activity per week for adults. It is important to note that this pattern can be attributed to the institutionalization of physical training in the context of the training course, as pointed out by Boçon (2015) and Medeiros (2019), who highlight the centrality of physical activity in police training. Considering the distribution among the different levels of intensity as light, moderate and intense, our data suggest a varied and comprehensive practice, reflecting a structured physical training routine.

To our knowledge, there is a scarcity of studies that investigate in detail the different levels of physical activity intensity during the Officer Training Course. One of the few available works is that of Vasconcelos Junior et al. (2025), who analyzed military police officers of Espírito Santo and sought to understand the relationship between stress symptoms, handgrip strength, and performance in police shooting. Although they did not directly examine the time of physical activity at different intensities, the authors highlighted the presence of stress symptoms in a relevant portion of the sample, suggesting possible psychophysiological implications for the functional performance of these military personnel. Our data were similar to the values reported by Vasconcelos Junior et al. (2025) of physical activity among military police from MPES, classified as active or very active. These findings suggest that the regular practice of physical exercise during the Officer Training Course provides a high physical demand, consistent with the institutionalized training routine.

Although the study did not perform specific statistical analyses between the levels of physical activity and psychological indicators, the authors highlight that physical activity plays a relevant role in promoting general health and may contribute to the reduction of stress-related symptoms. This association has already been widely documented in other studies, which demonstrate that the regular practice of exercises is related to improvement in mood, reduction of anxiety and depression, in addition to promoting emotional balance and the quality of life of practitioners (De Mello et al., 2013; Wiles et al., 2012; Souza et al., 2013). From this perspective, Vancini et al. (2018) demonstrated that in addition to its effects on physical fitness and body composition control, the high level of weekly physical activity observed also has positive implications in the psychophysiological domain, contributing to the regulation of stress, anxiety, and cognitive function. Thus, together, these findings reinforce the importance of maintaining regular physical training programs in the military environment, not only as a functional requirement but also as a strategy for promoting health and overall performance.

Additionally, the analysis of the participants’ physical performance in the PFT tests revealed satisfactory levels of fitness, reflecting a physical preparation compatible with the institutional requirements of the Military Police of Espírito Santo (PMES, 2023). The best average performances were observed in the push-up and rower sit-ups tests, indicating good localized muscular endurance of the upper limbs and trunk. These results are in line with the criteria established in the Manual for the Application of the Physical Fitness Test of the MPES (2023), which emphasizes the use of internationally validated protocols and the attribution of a score from 0 to 10 for measuring physical fitness in different age groups.

High levels of physical activity not only favor muscle conditioning and body weight control but also act positively on the modulation of stress, anxiety, and cognition (Vancini et al., 2018). Studies such as those by Boçon (2015) and Medeiros (2019) also point out that, in military training courses, there is a tendency for better performance in strength and localized muscular endurance capacities, while the indices of aerobic endurance and agility show greater variation among participants. In this sense, the findings reinforce the importance of a balanced and individualized physical planning, which contemplates the development of all physical capacities required in the performance of police activity, with a special focus on aerobic endurance and agility, fundamental components in real operational occurrences.

Considering the analysis of handgrip strength, it is worth mentioning that although this analysis is frequent at the international level (Simas et al., 2022; Orr et al., 2021; Canetti et al., 2024; Orr et al., 2017), in Brazil, to our knowledge, we have only one study (Vasconcelos Junior et al., 2025) that investigated this parameter in military police officers. According to Orr et al. (2017), a positive association is found between handgrip strength and performance in police tasks, indicating that recruits with underdeveloped handgrip strength face a higher risk of not being able to perform their occupational duties. In our study, similarly to Vasconcelos Junior et al. (2025), we did not find statistically significant differences between the dominant and non-dominant limbs, suggesting a well-developed functional symmetry among the participants. We believe that this similarity may be related to the ambidextrous nature of some operational activities, the standardization of military physical training, and a reflection of activity before the course rather than during course, thus more studies should be conducted to clarify it.

Another point that deserves attention refers to the evaluation of handgrip strength in the shooting position. To date, only Vasconcelos Junior et al. (2025) have evaluated this specific parameter in military police officer. Although our results were similar to those reported by these authors, the analysis is still preliminary and lacks additional studies for confirmation. However, as expected, the handgrip strength of the dominant limb in the shooting position was higher than that observed in standard position, which may indicate high neuromuscular activation in contexts that require precision and muscle tension (Canetti et al., 2024). In addition, Orr et al. (2017) evaluated active military police personnel showed that handgrip strength is positively associated with performance in occupational tasks, such as handling weapons and containment techniques.

Regarding musculoskeletal problems, there is robust evidence that low handgrip strength and weakness in the posterior trunk musculature increase the risk of chronic low back pain, especially in professions that involve load, prolonged posture, or heavy equipment, as is the case of military police officers (Range et al., 2023). Thus, the findings reinforce the need to incorporate integrated training strategies: strengthening the isometric strength of the upper limbs, scapular muscle endurance, and postural control, in order to prevent injuries and promote functional safety in operational situations. Additionally, the averages of isometric strength of the shoulder girdle muscles and deadlift show a general strength base compatible with the operational demands of the police function, although the high variability among individuals (CV > 25%) points to possible gaps in the homogeneity of physical preparation. These findings highlight the importance of training strategies that promote not only the development of maximum strength but also motor control and isometric endurance in specific contexts of action, such as the use of firearms, however, more studies are necessary to justify it.

Our results regarding performance in firearm shooting were similar to those reported by Vasconcelos Junior et al. (2025), indicating a satisfactory technical performance by the evaluated officers. The average execution time of the shots, the score obtained and the shooting performance coefficient demonstrate that, although the accuracy of the shots was high, the execution time was relatively high, which negatively impacted the overall performance coefficient. The coefficient of variation of 14.93% in this indicator also points to a considerable heterogeneity among the participants regarding the balance between speed and precision, a critical factor in real armed confrontation situations. Previous research, such as that by Nascimento et al., 2017, observed that the performance of police officers in shooting can be compromised after exposure to physical effort, especially when there is a drop in the capacity for concentration and fine motor control. However, in the present study, the technical performance was maintained within satisfactory parameters, possibly due to familiarity with the exercise, intensive prior training, and the use of Personal Protective Equipment (PPE), which provides greater emotional stability during the execution of the task. Furthermore, considering that most participants already had previous experience with weaponry before the Officer Training Course, it is plausible that this experience contributed to maintaining a high level of accuracy, even in the face of the course’s demands. Still, the discrepancy between precision and speed reinforces the need for training that simulates real operational scenarios, in which reaction time can be decisive for the survival of the officer or third parties.

When correlating the anthropometric, strength, mood state, and physical fitness parameters with the performance in firearm shooting, it was observed that specific aspects of body composition and muscle strength significantly influence the performance of the military personnel. The shooting score showed a positive correlation with body mass and BMI, as well as with fat mass. Although at first glance these findings may suggest that overweight benefits performance, it is necessary to interpret them in light of the body composition observed in the sample. As discussed earlier, although the average BMI indicates overweight, the low body fat percentage and good metabolic indicators point to a high proportion of functional muscle mass, typical of military personnel in training. This reinforces the limitations of BMI as an isolated indicator and suggests that greater body mass, when associated with strength and motor control, can contribute to greater postural stability and weapon control, favoring accuracy in shooting.

Dominant handgrip strength showed a statistically significant correlation with the shooting score, aligning with the literature that highlights the importance of isometric strength of the upper limbs for tasks that demand fine control and stability, such as the use of firearms (Canetti et al., 2024). This data also dialogues with the previous evaluation of the physical tests, which revealed good performance in exercises of localized muscular endurance of the upper limbs, especially in push-ups. The grip strength in the hand used in shooting position also showed a significant correlation with the score, suggesting that specific strengthening of this region can optimize technical performance. Such findings gain relevance when considering the high inter-individual variability in global strength levels, identified in the deadlift and scapular strength tests, indicating the importance of individualizing physical training with a focus on operational functionality.

Similarly to our study, Canetti et al. (2024) and Muirhead et al. (2019) suggest a correlation between shooting accuracy and grip strength. However, caution is needed when analyzing these findings. Several studies (Simas et al., 2022; Orr et al., 2021; Canetti et al., 2024; Muirhead et al., 2019; Vasconcelos Junior et al., 2025) have suggested more research aimed at investigating the relationship between shooting accuracy and handgrip strength for a better understanding of these parameters. However, studies have shown that handgrip strength, hand size (Canetti et al., 2024; Muirhead et al. 2019; Orr et al., 2021; Vasconcelos Junior et al., 2025), and the dimensions of the weapons used (Canetti et al., 2024; Muirhead et al. 2019; Vasconcelos Junior et al., 2025) can significantly impact shooting accuracy. In addition, Orr et al. (2021) propose the existence of a V-shaped curve between handgrip strength, accuracy, wingspan, and aim, with the standard size of the firearm grip acting as an intervening factor. Likewise, Vasconcelos Junior et al. (2025) identified that handgrip strength significantly affected the shooting ability of police officers, indicating that those with lower grip strength had fewer hits while using a firearm. It is worth mentioning that (Orr et al., 2021) demonstrated that a grip strength development ranging from 80 to 125 pounds (approximately 36.3–56.7 kg) was necessary to achieve approximately 85 to 95% shooting accuracy. Thus, it is possible to consider that our result may be associated with grip strength, since average forces for the dominant-, non-dominant hand and dominant hand in the shooting position were demonstrated. However, curiously, our results were different from the study by Vasconcelos Junior et al. (2025), who also investigated Officer Training Course students, using the same weaponry (Glock 0.40) and the same shooting condition (passive and aimed), indicating possible variability, and guaranteeing the need for more studies to clarify these findings. Furthermore, it is essential to conduct additional research using diverse scenarios, incorporating various types and weights of firearms under different shooting conditions. Thus, it is important to report that studies that have proposed to evaluate the parameters of firearm shots by Brazilian police officers are scarce (Vasconcelos Junior et al., 2025; Nascimento et al., 2017). Moreover, researchers address different scenarios, which makes it difficult to accurately evaluate the results; for example, Nascimento et al., 2017 evaluated the accuracy of police shots following a physical effort protocol, Nascimento Neto et al. (2015) investigated the effects of transcranial direct current stimulation on shooting accuracy, while Vasconcelos Junior et al. (2025) investigated shots in the passive and aimed condition.

From a psychophysiological point of view, vigor-one of the subscores of the mood state assessed by BRUMS—was the only emotional factor that positively correlated with the shooting score. This finding is particularly relevant in the context of the literature that associates the psychological profile with performance under pressure (Benevides-Pereira, 2010), reinforcing that individuals with greater vital energy or even motivation tend to maintain better performance in critical tasks, even under stressful conditions. This correlation is even more expressive when considering the high mood disturbance index identified in the sample, which could predispose to performance drops, but was, in part, counterbalanced by the good level of vigor observed. We believe that this balance may have been sustained by the high weekly load of physical activity of the participants, which is higher than the recommendations of the WHO (2020), which, as Vancini et al. (2018) argue, contributes to the regulation of mood and cognitive function, but more studies should be conducted to clarify this.

Finally, similarly to Vasconcelos Junior et al. (2025), the absence of significant correlations between the levels of physical activity (moderate, intense, and total) and shooting performance may be related to the fact that, despite being essential for general health and physical readiness, these indicators do not capture the neuromuscular specificity required in the use of firearms. This highlights the importance of integrating general physical training with specific shooting simulations, under conditions close to operational ones, as advocated by Nascimento et al. (2017). Together, the findings suggest that performance in the use of lethal force does not depend only on physical vigor or previous experience, but on a convergence between functional body composition, specific strength, balanced emotional state, and regular technical training, reinforcing the need for integrated programs for training and maintenance of armed proficiency.

The central finding in this study was the role of handgrip strength in firearm proficiency, indicating that localized isometric strength is a significant predictor of firearm accuracy. This observation aligns with international evidence (Canetti et al., 2024; Muirhead et al., 2019; Orr et al., 2021), which highlights the importance of grip force for stabilizing the firearm, maintaining control over the trigger, and reducing involuntary tremors during shooting. Importantly, our data extend these findings to the Brazilian context, where research remains scarce. Unlike many previous studies conducted in laboratory settings, we evaluated police officers wearing operational uniforms, equipped with personal protective equipment, and using the Defensive Shooting for Life Preservation method, a protocol designed to replicate the pressures of field operations. This enhances the ecological validity of our findings and demonstrates that the influence of grip strength persists even under more realistic and complex conditions.

An important contribution of this study lies in the psychophysiological dimension of performance. Among the mood states evaluated with the BRUMS questionnaire, vigor was the only factor significantly correlated with shooting score. Vigor represents a state of energy, motivation, and mental alertness, often considered the positive pole of psychological readiness. In contrast, negative states such as fatigue, anger, or confusion did not show significant associations, although their average levels were elevated in our sample. This finding suggests that while police officers may experience high psychophysiological demands and stress during training, the presence of vigor acts as a buffer, sustaining technical performance. Prior studies (Baldwin et al., 2022; Taverniers et al., 2010) have also indicated that emotional regulation and psychological resilience are critical for decision-making and fine motor control under pressure. Our study adds to this literature by demonstrating that vigor is not merely a psychological marker, but a factor with measurable influence on operational accuracy. From a training standpoint, this underscores the importance of programs that integrate psychological preparation—such as resilience training, stress inoculation, and mental conditioning—alongside physical and technical practice.

Some limitations in the present study deserve to be mentioned, especially due to the risk of erroneous generalizations. Among the limiting factors are: the sample size and the predominance (composed of men); the evaluation of physical activity carried out through self-administered questionnaires, which may have introduced significant variability in the data; the analysis focused on military personnel not primarily involved in operational activities; the evaluation of shooting accuracy in an educational environment and the implementation of shooting training immediately before the evaluation.

In conclusion, our data suggest that although physical conditioning is widely recognized as essential for the health, readiness, and performance of police officers, there remains limited evidence regarding how specific components of fitness and psychological state influence operationally relevant tasks such as shooting accuracy and speed. By integrating assessments of anthropometric parameters, fitness tests, grip strength, and mood states with a standardized shooting evaluation conducted under realistic conditions, this study makes an important contribution to understanding the determinants of firearm proficiency in Brazilian police forces. However, it is important to note that we believe firearm proficiency should be trained through specificity, in other words, by practicing with the firearm itself.

## Data Availability

The raw data supporting the conclusions of this article will be made available by the authors under reasonable request.
